# Impact of *Paracoccus* sp. EGY7 carotenoids on triple-negative breast cancer cells: invitro study

**DOI:** 10.1186/s13568-025-01825-5

**Published:** 2025-02-07

**Authors:** Karim Abdelazim, Ahmed Hussein, Sherine N. Khattab, Shaymaa Essam El Feky, Nehad Noby

**Affiliations:** 1 Department of Biotechnology, Institute of Graduate Studies and Research, Alexandria University, Alexandria, Egypt; 2 Chemistery Department, Faculty of Science, Alexandria University, Alexandria, Egypt; 3 Radiation Sciences Department, Medical Research Institute, University of Alexandria, Alexandria, Egypt

**Keywords:** Anticancer, Antioxidants, Carotenoids, Xanthophylls, *Paracoccus* sp., Zeaxanthin.

## Abstract

This study investigates the effect of *Paracoccus* sp. EGY7 carotenoids on the triple-negative breast cancer cell line (MDA-MB-231). The bacterial strain was isolated, and its carotenoids profile was analyzed via HPLC-DAD-MS. Cytotoxicity, migration tests and the expression of *BAX* and *BCL-2* at the gene and protein levels were carried out to evaluate the therapeutic efficiency of the produced carotenoids. Molecular docking analysis estimated the binding affinity between zeaxanthin and BCL-2 protein. Chromatographic analysis revealed zeaxanthin as the major carotenoid (48.3%). The extract exhibited significant cytotoxicity against MDA-MB-231 cells with an IC_50_ of 1200 µg. It notably reduced cell migration, with wound closure percentages of 37.50% and 79.17% for the 600 µg group, while the percentages were 12.50% and 53.50% for the 1200 µg group, compared to 71.67% and 95.67% for the control at 24 and 48 h post-treatment, respectively. The extract induced apoptosis, as evidenced by significantly increased *BAX/BCL-2* gene expression ratios at 600 and 1200 µg (*p* < 0.05). Western blotting showed increased BAX protein expression at 600 and 1200 µg compared to the control group (*p* < 0.001), and significantly lower BCL-2 protein expression (*p* = 0.000005 for 1200 µg and *p* = 0.0001 for 600 µg). Docking analysis indicated a strong affinity of zeaxanthin to BCL-2 (ΔG = -9.773241 kcal/mol) compared to obatoclax (ΔG = -7.419345 kcal/mol).* Paracoccus* sp. EGY7 carotenoids are a promising anticancer agent against MDA-MB-231 cells. They effectively promote apoptosis and prevent metastasis, crucial for disease advancement in cancer cells.

**Supplementary Information:**

The online version contains supplementary material available at 10.1186/s13568-025-01825-5.

## Introduction

Despite the substantial treatment advances in cancer over the years, it remains one of the world’s leading causes of death, with 10 million deaths expected from the disease in 2020 (Sung et al. 2021). The need for drugs that destroy cancer cells with a therapeutic index that is safely tolerated by the patient is the central problem in developing anticancer drugs. Historically, natural products (NPs) of plants or microbial origins have played a critical role in treating different health problems, especially cancer and infectious disorders. Owing to their structural complexity and chemo-diversity, NPs have revealed different bioactive characteristics with significant pharmacological advances for treating different types of cancer (Celedón and Díaz 2021).

Carotenoids are a class of NPs that have proven great potential for treating several diseases such as cataracts, atherosclerosis, multiple sclerosis, macular degeneration, and various types of cancer (de Morais et al. 2015; Sinha et al. 2022). Their health benefits are mainly attributed to their immune system enhancement, antioxidant and anti-inflammatory activities (Bhatt and Patel 2020). Carotenoids are synthesized by a wide variety of plants, algae, fungi, and bacteria and are responsible for the bright yellow, orange, and red colors found in these organisms (Bartlett 2013). Structurally, carotenoids are isoprenoid derivatives, biosynthesized through a head-to-tail condensation of two C20 geranylgeranyl diphosphate molecules constituting a C40 carbon chain, which is the main backbone of most known carotenoids (Maoka 2020). Other rare, longer carotenoids (C50), like bacterioruberin, are derived by the addition of two isoprene units (C_5_) to the C40 chain (Yang et al. 2015).They are synthesized in specific types of bacteria as an adaptation strategy under different stress condition (Hegazy et al. 2020; Noby et al. 2023).The long conjugated double bond system of the polyene chain constitutes the carotenoid chromophore, which is responsible for the coloration or pigmentation of carotenoids. Additionally, the resonance-stabilized intermediate produced by this lengthy polyene chain allows for an effective quenching of reactive oxygen species (ROS) (Namitha and Negi 2010). In this essence, bacterioruberin has gained significant interest because of its superior antioxidant activity.

Carotenoids are divided into two classes based on their chemical structure. (i) Xanthophylls, which are oxygenated carotenoids like zeaxanthin, cryptoxanthin, and lutein; and (ii) Carotenes, which are pure hydrocarbons without functional groups like α-carotene, β-carotene or lycopene (Maoka 2020). Generally, carotenoid synthesis is a multistep process controlled by gene cassettes. It starts with the formation of phytoene, a C40 intermediate and the first committed backbone in carotenoids structure. The enzyme phytoene dehydrogenase, which is encoded by the *crtI* gene, then dehydrates phytoene, yielding lycopene. The process is then completed by cyclizing lycopene via b-cyclase, encoded by the *crtY* gene to form β-carotene. In some species, the synthesized β-carotene molecule is further oxygenated to form xanthophylls either by β-carotene hydroxylase or ketolase encoded by *crtZ* and *crtW*, respectively (Lee and Kim 2006).

Bacteria held great promise in carotenoid production compared to other known sources. They produce a wide variety of carotenoids using simple nutritional media. In addition, their high growth rate and ease of cultivation, and manipulation have made them an ideal source of natural pigments (Ram et al. 2020). The genus *Paracoccus* is one of the main producers of rare xanthophyll such as astaxanthin, canthaxanthin, adonirubrin, and adonixanthin(Honda et al. 2020). Phylogenetically, this genus is part of the phylum Proteobacteria, class Alphaproteobacteria, order Rhodobacterales, family Rhodobacteraceae. The name *Paracoccus* was developed from the words “Para” (Greek for alongside or like) and “coccus” (Latin for grain or fruit). The genus harbors more than 96 recognized species (Cui et al. 2022; Davis et al. 1969), however, only a limited number of *Paracoccus* species are able to synthesize carotenoids, e.g. *P*. *carotinifaciens* (Tsubokura et al. 1999) *P. marcusii* (Harker et al. 1998) d *haeundaensis* (Lee et al. 2004). The expression differences in the *crtZ* and *crtW* genes result in unique profiles of the synthesized carotenoids among *Paracoccus* species. For example, *Paracoccus zeaxanthinifaciens* R-1534 produces zeaxanthin as the major carotenoid (Pasamontes et al. 1997), while astaxanthin represents the major carotenoids in *Paracoccus carotinifaciens* (14) and *Paracoccus haeundaensi* (12).

In recent years, the biological functions of microbial carotenoids have drawn a great attention in cancer research (López et al. 2023; Sinha et al. 2023; Tanaka et al. 2012). Many carotenoids have shown great potentiality as anticancer agents. For instance, bacterioruberin extracted from the halophilic archaeon *Natrialba* sp. M6 exhibited potent apoptotic and antiproliferative effects against breast, liver, and colon cancer cell lines (Hegazy et al. 2020). Similarly, carotenoids extracted from Halophilic Archaeon, *Haloarcula* sp. Showed high cytotoxicity on breast cancer cell line with IC50 = 0.0625 mg/mL (Shahbazi et al. 2023).Bacterial carotenoids have also shown potent anticancer activity. Staphyloxanthin pigment extracted from *Staphylococcus gallinarum* showed potent anticancer effects with an average IC50 of 6.5 µg /ml against several cancer cell lines like A549 Lung carcinoma, mucus skin melanoma (B16F10), and Ehrlich ascites carcinoma. While the pigment bore low toxicity with IC50 of 52.24 µg /ml towards the non- cancerous human fibroblast cell line (Barretto and Vootla 2018).

A significant inverse relationship between carotenoid intake and the incidence of numerous cancer types, such as breast (Eliassen et al. 2012), colorectal (Jung et al. 2013), and lung cancer (Takata et al. 2013) has also been shown in several case-control, cohort, and randomized trials. The exceptional antioxidant properties of carotenoids orchestrate their modulation of oxidative stress, which is considered the major cause of cancer prognosis. Moreover, numerous in vitro and in vivo studies have shown that carotenoids have a significant impact on reducing the progression of cancer by influencing the phosphorylation and activation of major signaling kinase (Kavitha et al. 2013), apoptosis (Dos Santos et al. 2018), angiogenesis (Şahin et al. 2012), metastasis (Yang et al. 2012), and multidrug resistance (MDR) (Eid et al. 2012).

In this respect, the introduced research study examines the carotenoids profile of the isolated strain, *Paracoccus* sp. EGY7. The apoptotic and antimetastatic effects of the extracted carotenoids are tested against the triple negative breast cancer cell line (MDA -MB-231 cells). To the best of our knowledge, the carotenoids extract of *Paracoccus* bacteria haven’t been tested before against the highly invasive and drug resistant MDA-MB-231 cells.

## Materials and methods

### Growth media

PY medium (10 g L^− 1^ peptone, 5 g L^− 1^ yeast extract, 5 g L^− 1^ NaCl), pH 7.0, was used in isolation step. PY medium was solidified with 1.5% agar to form PA medium.

PYT broth (10 g L^− 1^ peptone, 5 g L^− 1^ yeast extract, 5 g L^− 1^ tryptone), pH 8.0, was used as the optimized medium for carotenoid production.

### Isolation and purification of pigment producing bacteria

Bacterial isolation involved collecting samples from various soil sources, which were first activated on PY medium for 18 h. The activated cultures underwent serial dilution in 0.9% saline, spread onto PA plates, and incubated at 30 °C. Colored colonies were selected, purified, and stored at -20 °C in 20% glycerol. Morphological and microscopic examinations were conducted on the isolates, followed by molecular identification using the 16 S rRNA approach (Eden et al. 1991). Genomic DNA was extracted using the DNA miniprep kit (D3024, ZYMO), and the full length of the 16 S rRNA gene was amplified via PCR using F27 and R1492 primers. The PCR product was sequenced and analyzed using BioEdit and BLAST n tools, respectively, with a phylogenetic tree constructed using MEGA X software, for further information, please refer to supplementary, Section 1.

### Pigment production, extraction, and quantification

A volume of 20 mL of PYT medium was inoculated with freshly activated colonies of the selected bacterial isolate and incubated overnight at 200 rpm, 30 ^o^C. One liter of the production medium (PYT) was sterilized and inoculated with 5% of the activated culture, then incubated under the same conditions for 48 h. At the end of the cultivation time, cell pellets were harvested via centrifugation at 2000 g for 15 min. The collected pellets were soaked in a mixture of methanol and ethyl acetate (1:1) for 2 h under shaking at room temperature until the cells were completely bleached. The cell debris was removed via centrifugation under cooling for 20 min at 2000 g. The obtained carotenoid extract was wrapped in aluminum foil and stored at -20 ^o^C. The extracted pigment was scanned in the range of 200–800 nm using a UV/Vis spectrophotometer. The total carotenoid concentration was estimated at the maximum wavelength (λ_470_) by applying an average extension coefficient reported by Liaaen-Jensen and Jensen, 1971 (Liaaen-Jensen and Jensen 1971).

### Correlation of growth pattern and pigment accumulation of *Paracoccus* sp. EGY7

Pigment accumulation pattern was correlated with cell dry weight during an incubation period of four days to estimate the maximum production time of the pigment. The isolate was activated as reported before, and then 5% of the activated culture (10^9^ CFU/mL) was inoculated into PYT broth. The culture was incubated at 30 ^o^C and 200 rpm. Samples were withdrawn from the culture at constant time intervals to quantify both pigment yield and cell dry weight. Each sample was subjected to total pigment extraction as previously described, and the remaining cells were dried at 50 ^o^C until reaching a constant weight. Pigment concentration was calculated in terms of (µg/g _cell dry weight_) according to the following equation.

$$\begin{aligned} e_\tau (X)= & \underset{{\epsilon } \in \mathbb {R}}{argmin}\int _{\mathbb {R}}(x-{\epsilon } )^2(\tau \mathcal {I}_{(x>{\epsilon } )}\nonumber \\ & +(1-\tau )\mathcal {I}_{(x<{\epsilon } )}) dF(X) \end{aligned}$$

A: Sample absorbance at maximum wave length.

V: Volume of the extract.

∑1%: Molar extension coefficient at a concentration of 1%.

W: Cell dry weight calculated in grams.

### Pigment characterization

HPLC-DAD chromatography, UV-visible spectroscopy, and LC/ESI-MS-MS spectrometry were used to characterize the extracted pigment. Chromatographic analysis of the extract was carried out using HPLC coupled to diode array detector. A volume of 50 µL of the prefiltered pigment extract was fed into a C_18_ reversed phase column (50 mm x 2.1 mm, 1.7 μm, Milford, MA01757, USA) with a flow rate of 0.2 mL/min. Separation of target compounds was carried out using a mixed mobile phase of methanol: acetonitrile (v/v 80:20). The eluted peaks were detected in the range of 200–600 nm while the chromatogram was built at 470 nm. Pigment composition was further investigated by mass spectrometry, where the separated peaks with readings in the visible regions were further examined on LC-MS-MS column under the same elution conditions. The spectrum was investigated in positive ionization mode in the range of 200–3000 m/z. Both HPLC-DAD chromatography and LC/ESI-MS-MS spectrometry were used to identify the obtained fractions.

### Preparation of MDA-MB-231 cell culture

Triple negative adenocarcinoma human breast cancer cell line MDA-MB-231 was purchased from the American Type Culture Collection (ATCC, Virginia, USA). Cells were cultivated in DMEM (Gibco, USA) and supplemented with 10% (w/v) FBS (Gibco, USA) and 1% antibiotics (100 U/ml streptomycin and 100 U/ml penicillin). The cells were cultured in 96 and 6 well plates according to subsequent investigations and incubated in a humidified atmosphere containing 5% CO2 at 37 °C and until they reached 75–80% confluence. After completion of the treatment protocols listed below, cells were harvested using 0.05% trypsin (Gibco, USA) and 0.02% EDTA.

### The neutral red uptake assay

MDA-MB-231 cells cultured in 96 well plates were treated with serial concentrations of *paracoccus* sp. EGY7 carotenoids extract (0, 37.5, 75, 150, 300, 600, 1200 µg) dissolved in 0.01% DMSO. After 48 h incubation, the culture medium was removed, and cells were rinsed with 200 µl of PBS. Then, 150 µl of 1x Neutral Red Staining solution was added to each well and incubated for 2 h. Afterword, the staining solution was removed and 250 µl of PBS was used to wash the wells. Subsequently, 150 µl of de-staining solution (50% ethanol, 1% acetic acid, 49% distilled water) was added to the wells, shaken for 20 min, then the O.D. was measured at 540 nm using the enzyme-linked immunosorbent assay (ELISA) microplate reader (Infinite F15 TECAN, Switzerland). The cell viability percentage was calculated using the equation: Viable cell % = (OD treated / OD untreated) × 100. The IC50 values, or the concentration of a chemical that inhibits 50% of the growth of cells relative to the untreated control cultures, were calculated using the percentages of cell viability.

### Scratch wound healing assay

A scratch wound healing assay was performed to assess the wound healing effect of pigment on MDA-MB-231 cells. Cells were seeded in 6-well plates and followed up until they formed a confluent monolayer. The media was removed, and the confluent monolayer was scratched with a pipette tip and rinsed with PBS to remove cell debris. Cells were then incubated in fresh media with different concentrations of pigment (0, 600, 1200 µg) and DMSO for 48 h. the migration of cells through the scratch was monitored and photographed 24 and 48 h after the incubation and images were analyzed using ImageJ software.

### Reverse transcription quantitative polymerase chain reaction (RT qPCR)

RT-qPCR was employed to quantify the expression of BCL-2 and BAX genes in MDA-MB-231 cells treated with varying concentrations (0, 600, or 1200 µg) of pigment and DMSO for 48 h. Total RNA was isolated using the Fast HQ Extraction kit (Intron Inc.), and cDNA synthesis was performed using the SensiFAST cDNA synthesis kit (Meridian Life Science Inc.). RT-qPCR reactions were conducted on a CFX Connect Real-Time PCR System (BioRad Inc.) using specific primers for BCL2, BAX, and GAPDH under defined cycling conditions. Results were analyzed using the 2-ΔΔCt method to determine relative gene expression levels, for further information please refer to supplementary, Section 2.

### Western blot analysis 

Western blotting was utilized to quantify BCL-2 and BAX protein levels. Total protein extraction was achieved using the ReadyPrep protein extraction kit (Bio-Rad Inc.), and protein concentrations were determined via the Bradford assay. Samples containing 20 µg of protein were loaded onto SDS-PAGE gels, separated, and transferred to nitrocellulose membranes. Membranes were blocked and incubated with primary antibodies (Novus Biologicals Inc.), followed by secondary antibody incubation and detection using the Clarity Western ECL substrate (Bio-Rad Inc.). Chemiluminescent signals were captured and analyzed using image analysis software for protein normalization against beta-actin for further information, please refer to supplementary, Section 3.

### Target proteins and ligands preparation

SwissDock software was applied for the docking analysis. The protein BCL-2, identified by the code 2W3L, was obtained from the PDB database. A collection of ligands, including zeaxanthin (PubChem CID: 5280899) and the standard drug Obatoclax (PubChem CID: 11404337), was assembled. The structures of these compounds were downloaded in SDF format from PubChem. Subsequently, the ligands were converted from SDF to MOL2 format and then imported into SwissDock software for analysis.

### Structure analysis

The UCSF Chimera analysis of the BCL-2 protein complex with the ligand zeaxanthin revealed key structural and interaction insights. The parameters used were an energy cutoff of -0.5, a minimum helix length of 3 residues, and a minimum strand length of 3 residues. This process identified 236 hydrogen bonds within the BCL-2 protein itself, which are vital for the stability of the protein’s three-dimensional structure. The contact analysis was conducted with an allowed van der Waals overlap threshold of 0.6 Å, reduced by 0.4 Å for hydrogen bonds, effectively making the threshold 0.2 Å for hydrogen bonds. Interactions between atoms within the same residue or within 4 bonds were excluded, while intra-molecule contacts were considered.

### Statical analysis

The Shapiro-Wilk test was carried out to assess the normality of the data. Upon confirming normality, an ANOVA test was conducted to compare the means across different groups, thereby determining if statistically significant differences exist among them. Subsequently, Tukey’s Honest Significant Difference (HSD) post hoc analysis was used to identify specific group pairs that exhibit significant differences. This analysis was performed with a 95% confidence interval and a significance threshold set at a p-value of less than 0.05. All statistical calculations were executed using R software version 4.2.2, ensuring precise and reliable results.

## Results

### Isolation and identification of pigment producing isolates

**Fig. 1:**
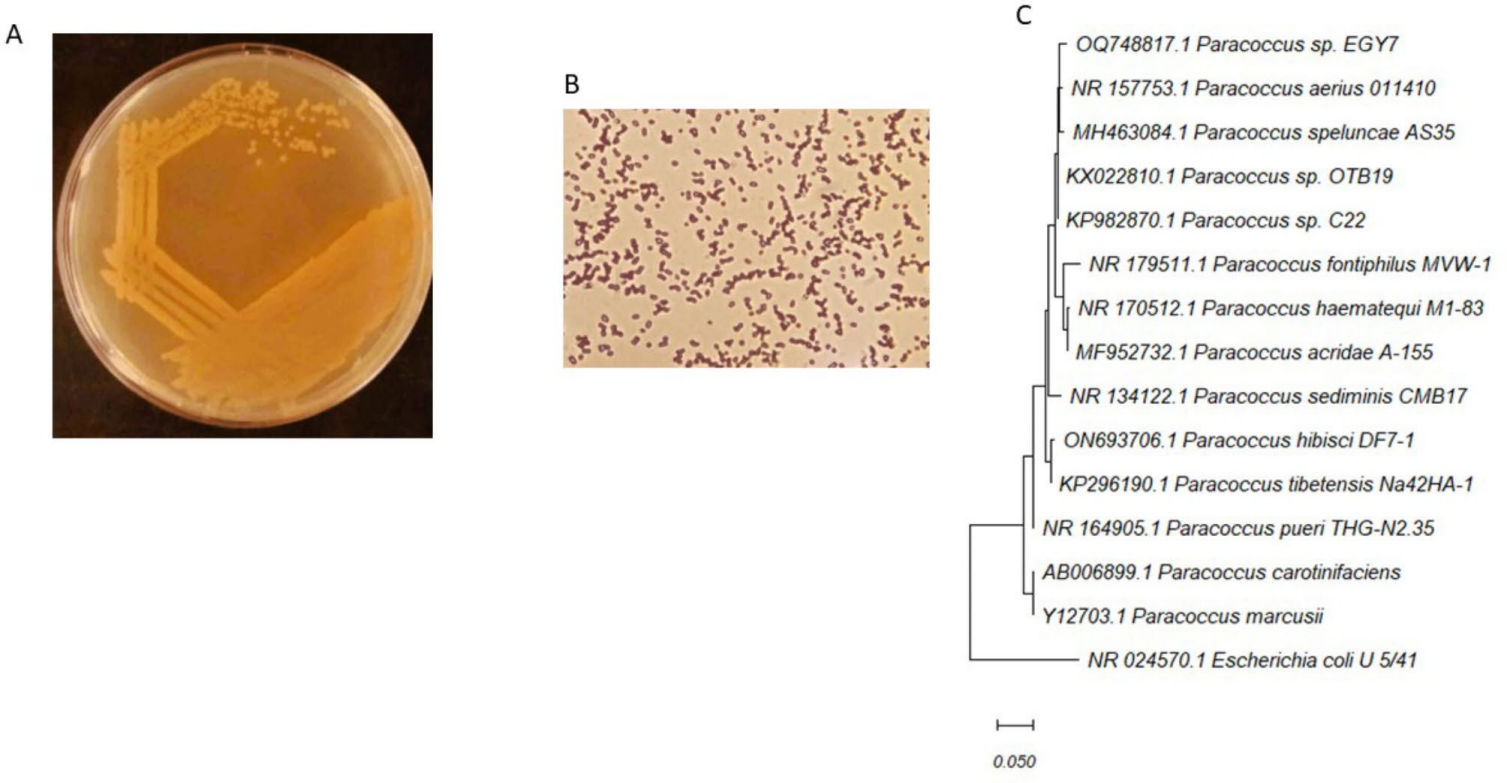
Identification of *Paracoccus* sp. EGY7. **A** Colony morphology of *Paracoccus* sp. EGY7 on PY medium. **B** Cell morphology under the light microscope. **C** Phylogenetic analysis of *Paracoccus* sp. EGY7 with closely related species using maximum likelihood. Accession numbers are displayed for each genus. Bootstrap values are shown as a percentage of 500 replicates. *Escherichia coli* was added as an outgroup

The isolation step resulted in three isolates with different hues. For safety considerations, the isolates were first subjected to molecular identification to select a proper carotenoid producing strain. Based on 16 S *rRNA*, *Paracoccus* isolate was chosen as a suitable and potent source of some rare xanthophylls. The isolate appeared orange on PY plates with rounded, smooth colonies (Fig. 1A). Cells under the light microscope appeared as short cocci (Fig. 1B). Taxonomic analysis of the isolate revealed its affiliation to *Paracoccus* sp. (Fig. 1C). The strain was named EGY7, and its sequence was deposited in the Genbank under accession number OQ748817.1. The strain was deposited in Egyptian Microbial Culture Collection EMCC under number EMCC-4081 Fig. 1.

### Production and identification of *paracocccus* sp. EGY 7 carotenoids

**Fig. 2:**
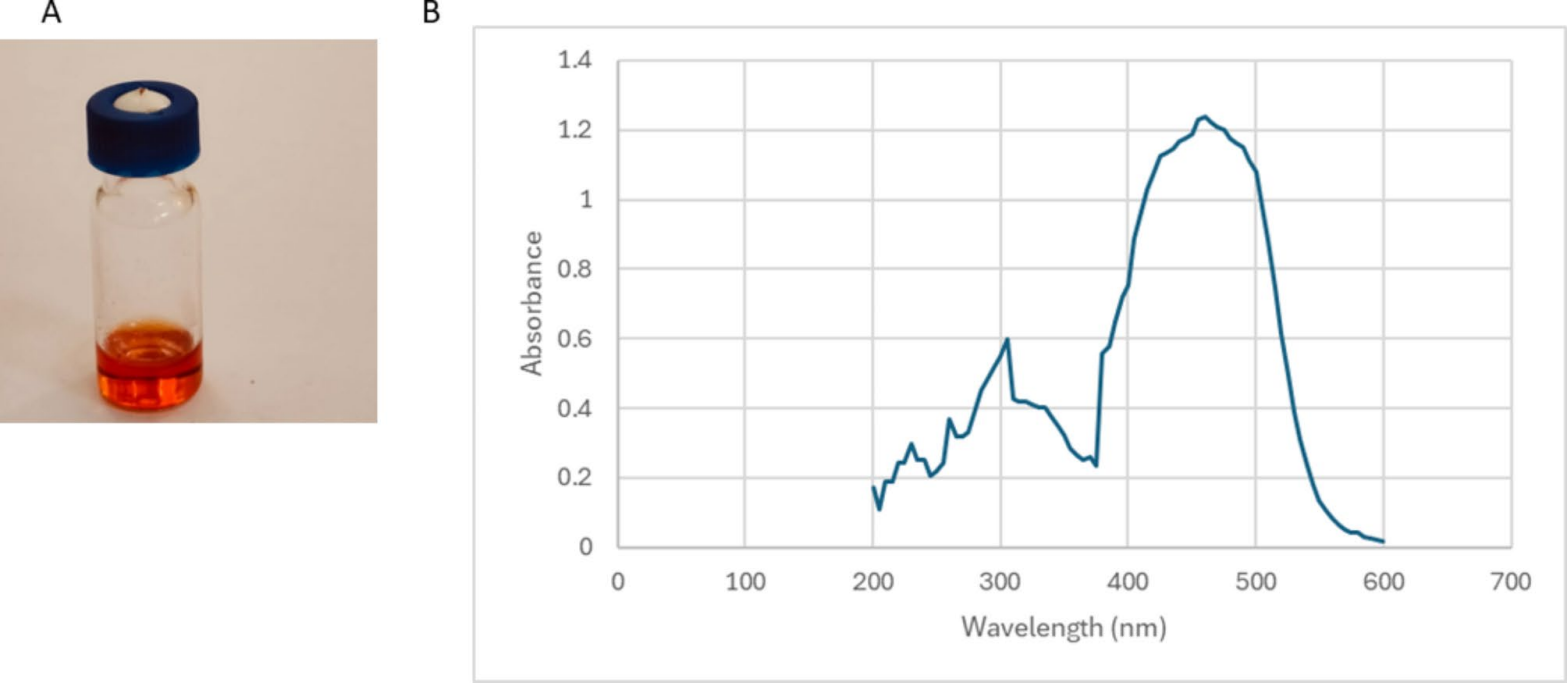
**A** Solvent extract of *Paraccoccus* sp. EGY7 pigment using methanol: ethyl acetate (1:1). **B** Spectrophotometric scanning spectrum of the extracted pigment illustrating the maximum absorption at 470 nm

The solvent extract of the pigment appeared reddish orange (Fig. 2A). The UV/Vis scanning demonstrated its maximum absorption point at 470 nm (Fig. 2B).

Pigment production pattern was plotted against cell dry weight over three days of incubation to estimate the maximum production time of the pigment. As shown in Fig. 1S, the pigment accumulation pattern of the isolate followed its growth pattern, where the pigment yield increased gradually from the onset of the log phase until it reached its maximum value at the beginning of the stationary phase. After 48 h., there was a noticeable decline in pigment yield parallel to the late stationery and decline phases (Fig. 1S), which can be attributed to pigment leakage outside dead cells Fig. 2.

### Analysis and characterization of EGY7 carotenoids profile

**Fig. 3:**
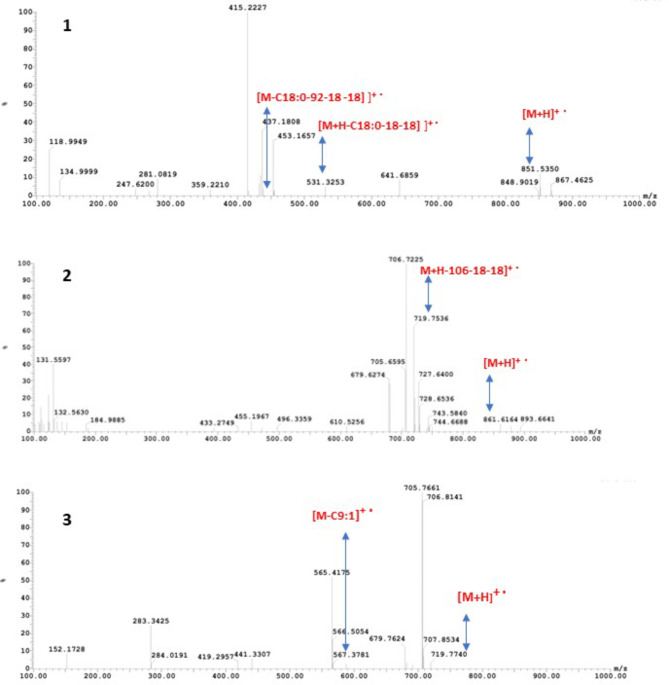
LC/ESI-MS/MS spectra of the three eluted peaks (Peak 1, 3, and 5) of EGY7 carotenoids. The predicted fragmentation products in each peak are added in red

The analysis of the UV-Vis chromatogram of the eluted peaks demonstrated that only peak 1,3, and 5 had readings in the visible region with maximal absorbance at 475,470, and 458, respectively (Fig. 2S). Based on the maximum absorption wavelength and the mass spectra of each fraction (Fig. 3), the extracted carotenoids were identified as antheraxanthin monoester, astaxanthin monoester, and zeaxanthin monoesters (Tables 1 and 2).

### The neutral red uptake assay

The data suggested that the pigment has a dose-dependent cytotoxic effect on MDA-MB-231 cells. Higher concentrations (1200 µg and 600 µg) significantly reduce cell viability, indicating higher toxicity. As the concentration decreases, the cytotoxic effect diminishes, with very low concentrations (75 µg and 37.5 µg) showing negligible impact on cell viability. This trend indicates that the pigment is safe at lower concentrations but becomes harmful with increasing the concentration. From the data, we observe that the concentration at which the cell viability is closest to 50% is 1200 µg.

The linear regression model indicated a strong negative linear relationship between pigment concentration and cell viability, with a highly significant decrease in viability as the concentration increases. The p-value for the concentration coefficient is 0.000281, which is much less than the typical significance level of 0.05. This means the relationship between concentration and viability is statistically significant (Fig. 3S).

### Gene expression statues of *BAX* and *BCL-2* after the treatments

The ANOVA test revealed that there is a statistically significant difference among at least one pair of groups (control, DMSO, concentration 600 µg, and concentration 1200 µg) based on their qPCR results in MDA-MB-231 cells. This suggested that at least one of the treatments has a different effect on gene expression compared to the others. The post-hoc Tukey test was conducted to examine the differences in fold change of *BAX/BCL-2* gene expression between the groups (control, DMSO, concentration 600 µg, and concentration 1200 µg).

**Fig. 4:**
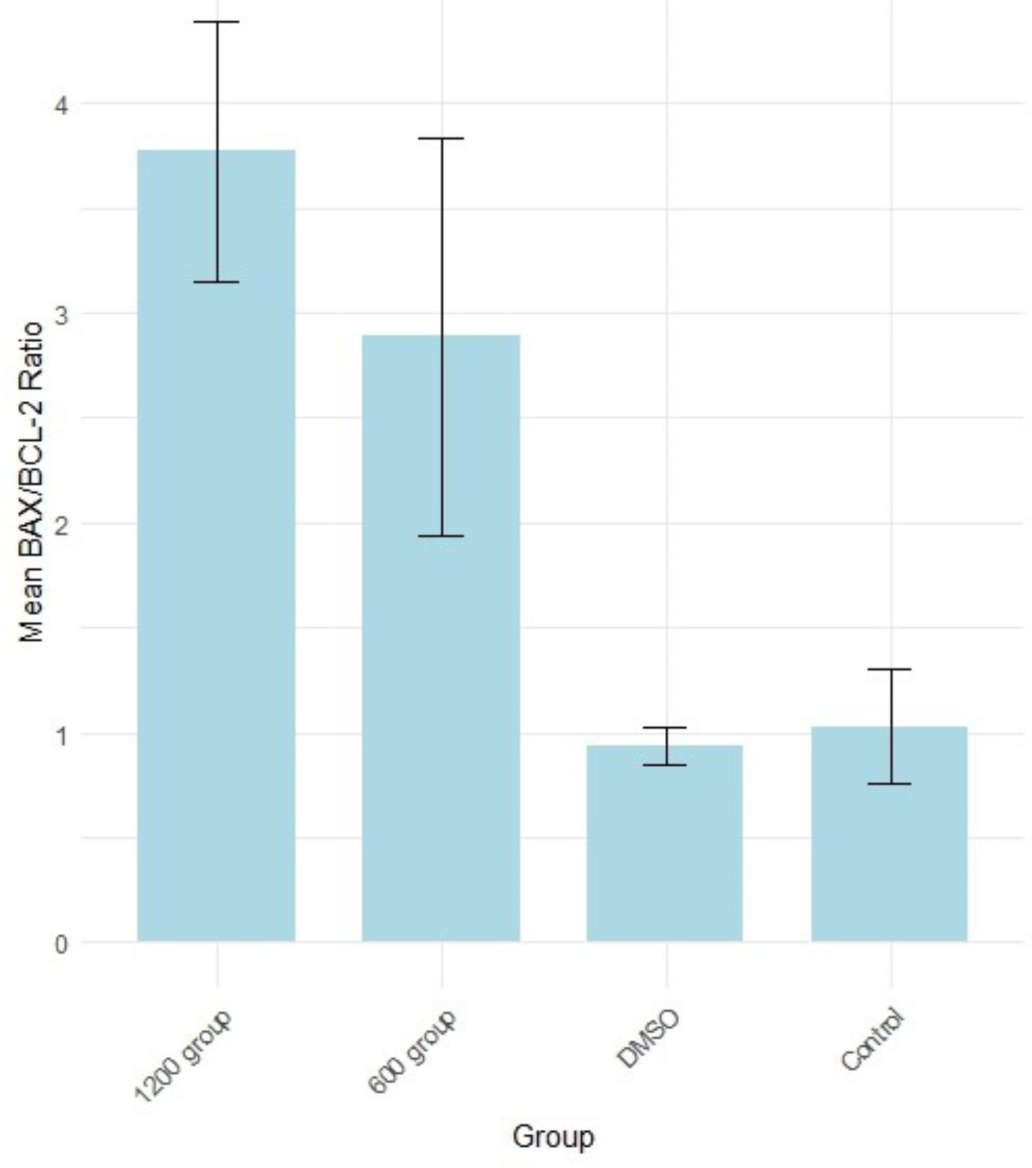
Mean *BAX/BCL-2* fold change

The gene expression analysis revealed a decrease in *BCL-2* gene expression and an increase in *BAX* gene expression. This resulted in significant increases in the *BAX/BCL-2* ratio gene expression at the 1200 µg concentration group compared to both the control (*p* = 0.0019536) and DMSO (*p* = 0.0015685) groups. Similarly, the 600 µg concentration group exhibited significantly higher gene expression ratios compared to the control (*p* = 0.0194848) and DMSO (*p* = 0.0149616) groups. However, no significant difference was found between the DMSO and control groups (*p* = 0.9970772), and there was no statistical difference between the 600 µg and 1200 µg groups (*p* = 0.3189472) (Fig. 4) (Table 1S).

**Fig. 5:**
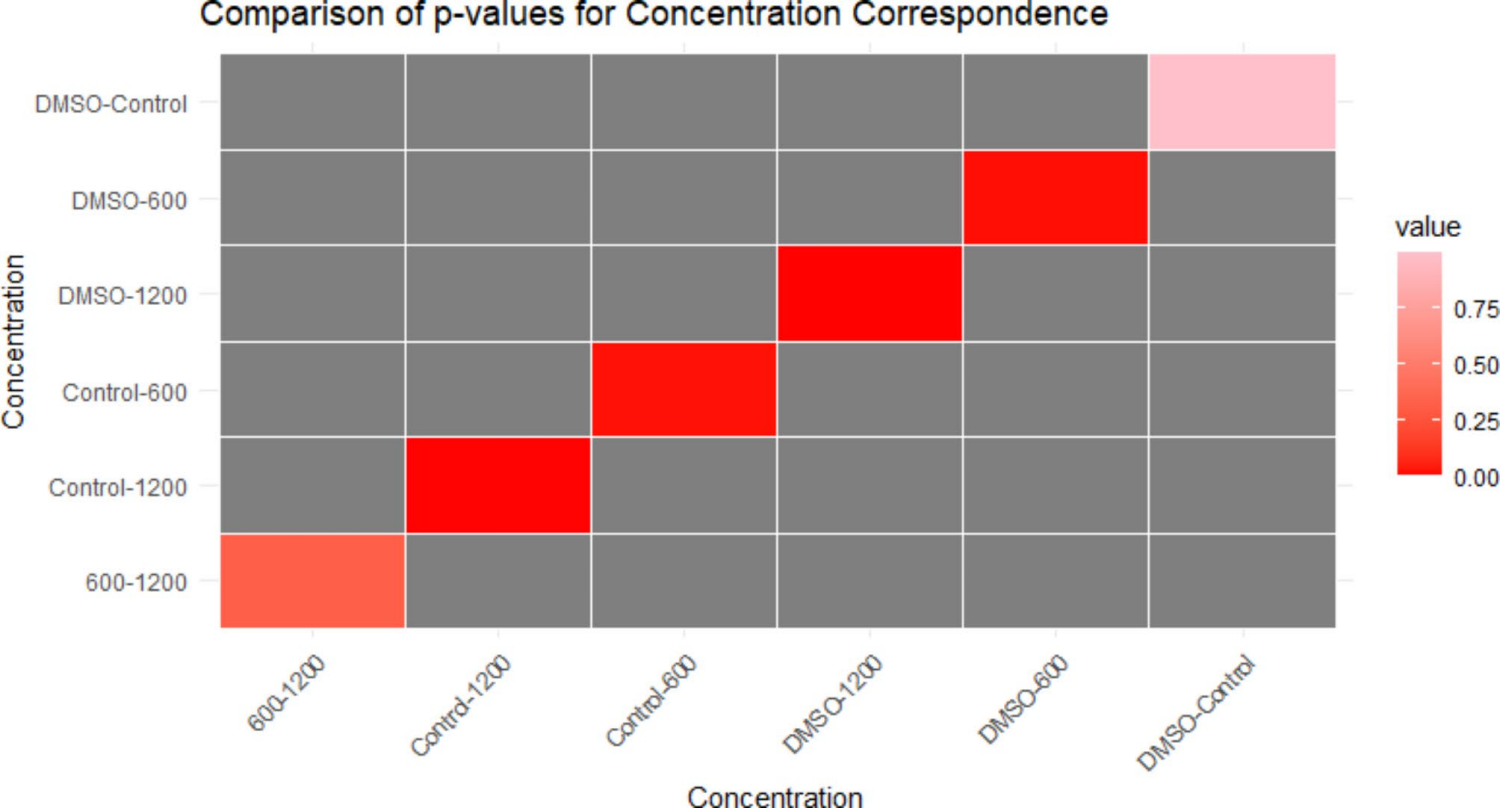
Significance Gradient: Heatmap of p-values

These findings underscore the substantial impact of pigment concentrations, particularly at 1200 µg and 600 µg, on *BAX/BCL-2* ratio gene expression in MDA-MB-231 cells. The notable increases in gene expression levels compared to both control and DMSO treatments suggested a distinct regulatory effect exerted by the pigment. Overall, these results provide valuable insights into the molecular mechanisms involved in modulating *BAX/BCL-2* gene expression by the tested pigment concentrations. The heatmap displays the p-values for each comparison, with darker shades of red indicating greater significance (Fig. 5).

### Comparison of BAX and BCL2 protein expression across treatment groups

The analysis of BAX protein expression revealed significant variations across different treatment concentrations (*p* < 0.001),

There was a pronounced difference between the 600 µg and 1200 µg groups (*p* = 0.0006), with the 1200 µg group exhibiting higher BAX expression. Similar significant disparities were found between the Control and 1200 µg groups (*p* = 0.0001), and between the DMSO and 1200 µg groups (*p* = 0.0001), both showing elevated expression levels in the 1200 µg group. Additional significant differences were observed between the Control and 600 µg groups (*p* = 0.0008), and between the DMSO and 600 µg groups (*p* = 0.0012), indicating higher expression in the 600 µg group.

**Fig. 6:**
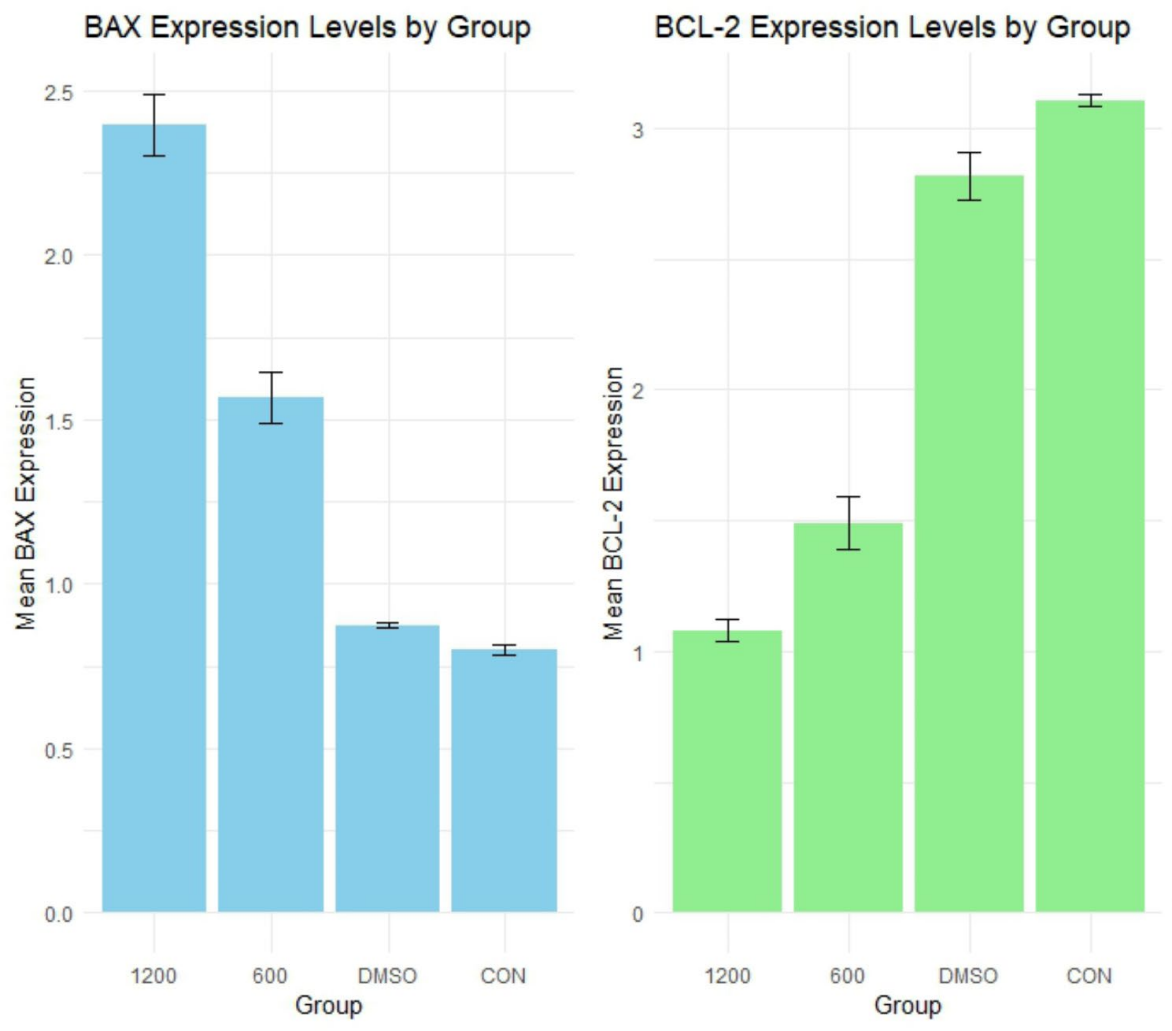
Comparison of BAX and BCL-2 protein expression

These results collectively suggested a clear trend of increased BAX expression with higher treatment concentrations, while the effects of DMSO and the Control were similar, showing no significant difference (Figs. 6 and 4S) (Table 2S).

The analysis of BCL-2 protein expression also revealed significant differences across various treatment concentrations (*p* < 0.001).

A significant difference in BCL-2 expression was observed between the 600 µg and 1200 µg groups (*p* = 0.0157), with the 1200 µg group showing lower BCL-2 expression. Similarly, there were marked differences between the Control and 1200 µg groups (*p* = 0.00005), and between the DMSO and 1200 µg groups (*p* = 0.00008), both indicating lower expression levels in the 1200 µg group. Furthermore, significant contrasts were noted between the Control and 600 µg groups (*p* = 0.0001), and between the DMSO and 600 µg groups (*p* = 0.0002), showing reduced expression in the 600 µg group.

These findings highlighted a consistent trend of decreased BCL2 expression with increasing treatment concentrations (Figs. 6 and 4S, Table 2S).

### Scratch wound healing assay

**Fig. 7:**
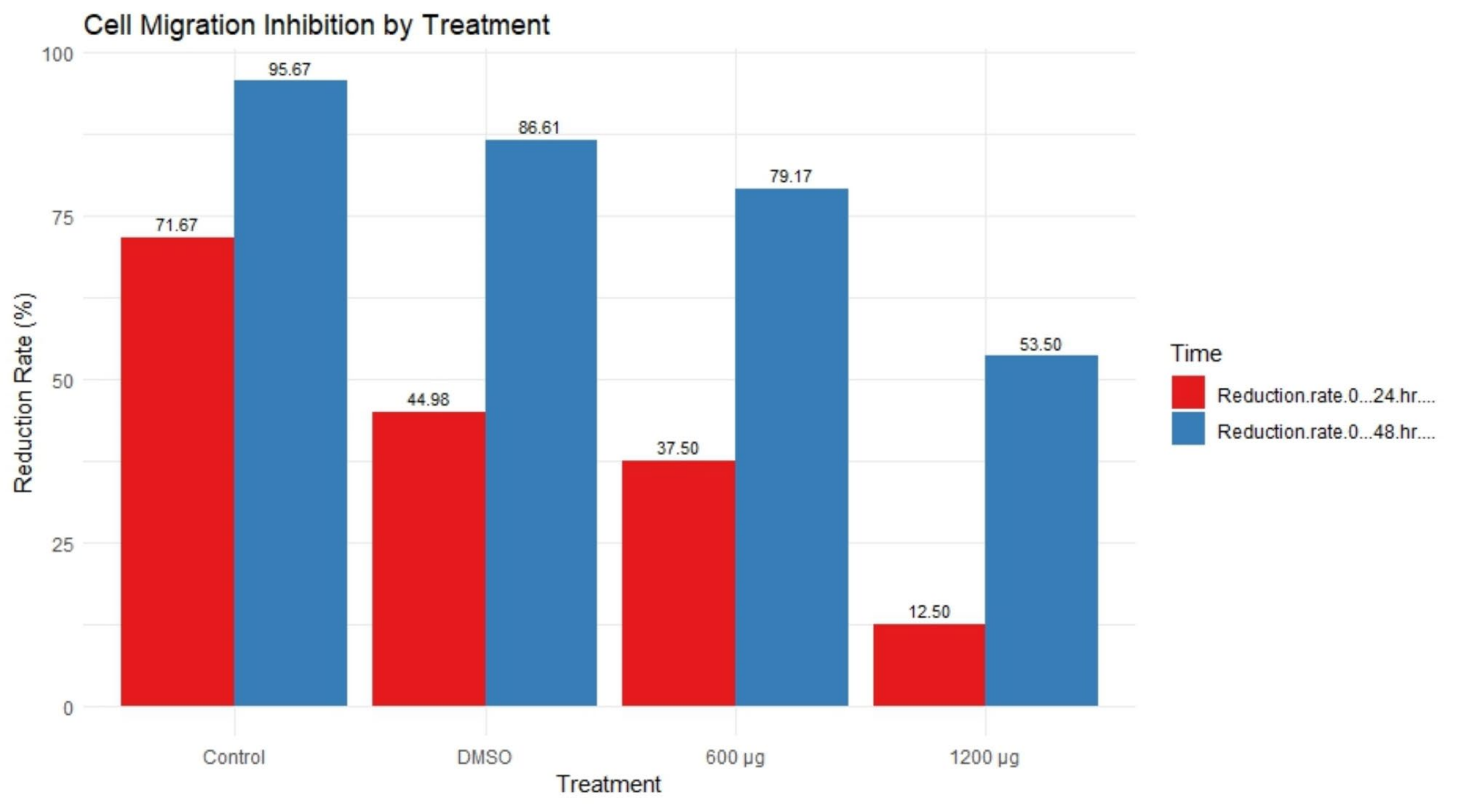
Cell migration inhibition by treatment

The migration assay results for MDA-MB-231 cells indicated that cell migration is significantly affected by the treatments in a dose-dependent manner. The control group, with no treatment, exhibited high reduction rates (71.67% at 24 h and 95.67% at 48 h), demonstrating robust cell migration. In contrast, the DMSO-treated group showed a moderate reduction in migration (44.98% at 24 h and 86.61% at 48 h), indicating some inhibitory effect of the solvent itself. With 600 µg of treatment, cell migration was further reduced (37.50% at 24 h and 79.17% at 48 h), suggesting a stronger inhibitory effect. The 1200 µg treatment resulted in the most substantial inhibition of migration (12.50% at 24 h and 53.50% at 48 h), highlighting a potent effect at this higher concentration. Overall, the data demonstrate that the treatment effectively inhibits MDA-MB-231 cell migration in a concentration-dependent manner, with higher doses leading to greater inhibition (Table 3S, Fig. 7).

### Molecular docking analysis

**Fig. 8:**
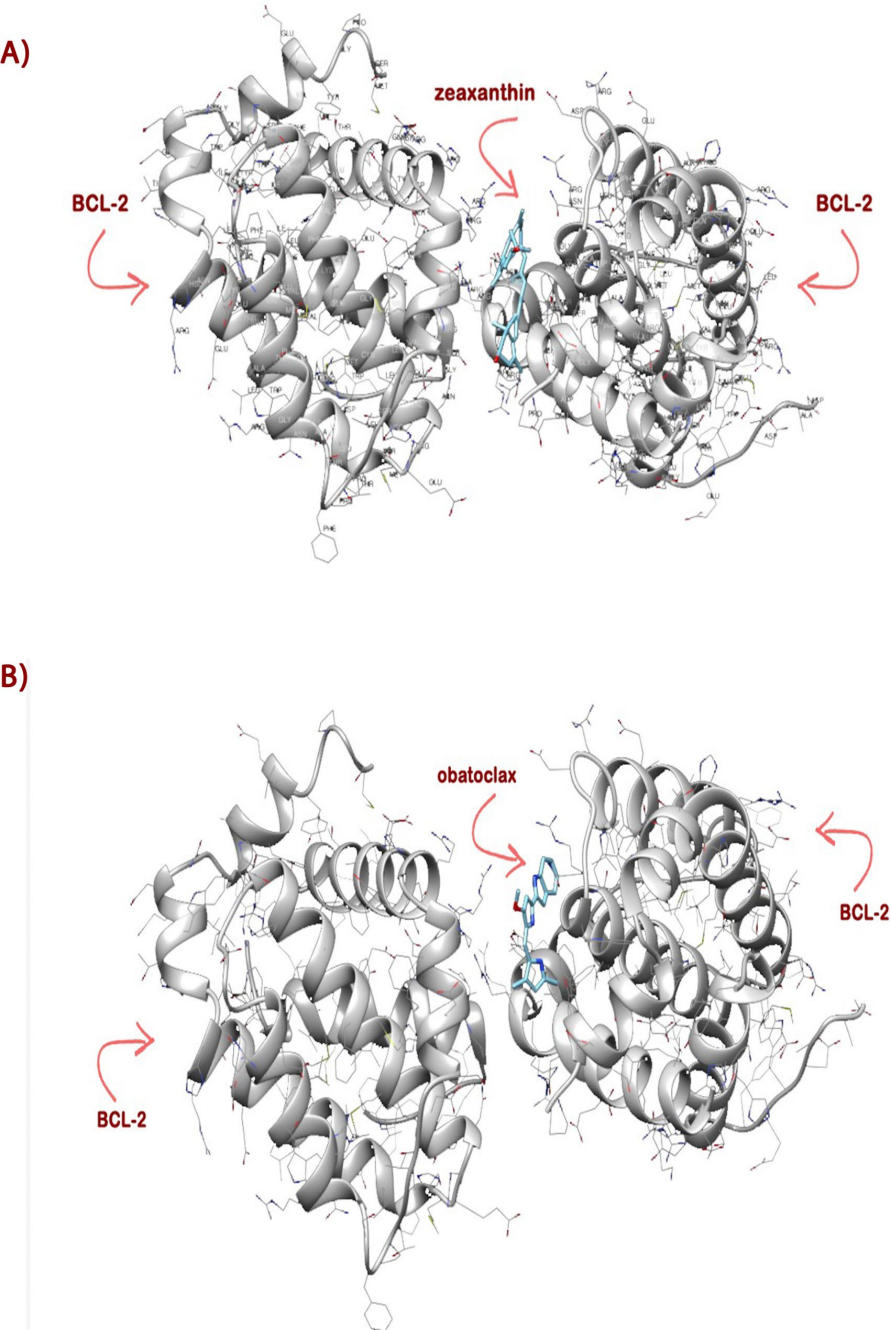
**A** Molecular docking of zeaxanthin and BCL-2. **B** Molecular docking of obatoclax and BCL-2

The molecular docking results for the interaction between the BCL-2 protein and the ligand zeaxanthin indicate a strong binding affinity. The key metrics provided are a FullFitness value of -2218.46 kcal/mol and an Estimated ΔG of -9.773241 kcal/mol. These values suggest a highly favorable and stable interaction between BCL-2 and zeaxanthin. The highly negative FullFitness value reflects the overall stability and favorability of the binding configuration in the docking simulation, while the estimated ΔG indicates a spontaneous binding process with a strong likelihood of occurrence under physiological conditions (Fig. 8A, Table 4S).

The molecular docking results for the interaction between the BCL-2 protein and the ligand obatoclax reveal a moderately strong binding affinity. The key metrics provided include a FullFitness value of -2203.04 kcal/mol and an estimated ΔG of -7.419345 kcal/mol. These values suggest a favorable and stable interaction between BCL-2 and obatoclax (Fig. 8B, Table 4S).

### Structure analysis

The structure analysis between BCL-2 protein and zeaxanthin revealed two significant interactions characterized by their van der Waals overlaps and distances. Specifically, an interaction between the hydrogen atom (HE) of arginine residue 65 and the carbon atom of serine residue 64 had overlaps of 0.705 Å and 1.995 Å. Another interaction was found between the hydrogen atom (HN) of arginine residue 65 and the carbon atom of phenylalanine residue 63, with overlaps of 0.621 Å and 2.079 Å. These overlaps exceed the allowed threshold of 0.6 Å, indicating strong contacts. These interactions suggest effective binding of zeaxanthin to BCL-2, potentially influencing its structural stability and function. These critical interaction points highlight pivotal sites for the ligand’s binding affinity and the protein’s conformational integrity.

## Discussion

Numerous carotenoids (CTDs) have been discovered to possess considerable ROS-scavenging activity (antioxidant activity), which is linked to cytotoxicity (antiproliferative activity) against cancer cells and thus could be employed as therapeutic and preventative agents for cancer (Ali et al. 2015; Ziech et al. 2010). This association is clear from the observations that an adequate content of consumed CTDs may inhibit the process of initiation, progression, and metastasis of cancer (Starska-Kowarska 2022). A diet containing CTDs provides many advantages and potentially allows the regulation of many cancer types such as head and neck cancer (HNC) and skin cancer (Black et al. 2020; Starska-Kowarska 2022). Carotenoids exert their function through different mechanisms and at different levels, for instance, they act as quenchers of singlet molecular oxygen, repress the formation of free radicals via inhibiting the autooxidation chain reaction, and create more stable molecules from hydroperoxides (Galasso et al. 2017).

The biological functions of xanthophylls like lutein, zeaxanthin, cryptoxanthin, capsanthin, astaxanthin, and fucoxanthin have received significant interest in cancer research in recent years. (Kotake-Nara and Nagao 2011) Their improved ability to quench free radicals is partly due to their higher polarities, which are attributed to the carbonyl (e.g., canaxanthin) or hydroxyl groups (e.g., zeaxanthin) present in the terminal rings of carotenoids (Dewanjee et al. 2021). Xanthophylls can be found in their free form or acylated with fatty acids (FAs), where mono- and polyhydroxylated xanthophylls commonly form monoesters or diesters. Generally, the esterification of carotenoids enhances their stability and protects them against photo-oxidation. The genus *Paracoccus* is one of the known producers of rare xanthophylls with a varied carotenoids profile depending on the producing species (Maj et al. 2020).

High performance liquid chromatography is the main technique used for carotenoid identification. Carotenoids are preferably separated by reversed-phase high performance liquid chromatography (RP-HPLC) because of its high selectivity and ease of manipulation (Bijttebier et al. 2013). The spectral characteristics of carotenoids can be estimated through photodiode array detector (PDA). However, because PDA detectors do not yield much structural information, it can be exceedingly challenging or even impossible to identify an unknown carotenoid component based only on its PDA spectrum. In this respect, data obtained by mass spectrometric (MS) detectors in terms of molecular weight and fragmentation pattern are much more helpful and can facilitate the identification of the carotenoid profile. Because of the oxygen containing functional groups in xanthophylls, their fragmentation results mainly in the formation of protonated molecules (MH + ions). Water loss fragments are usually formed during the fragmentation of protonated molecules, consequently [MH-H_2_O] + and [MH-2H_2_O] + fragments are significantly detected (Bijttebier et al. 2013). typical carotenoid fragments corresponding to ester cleavage [MH-FA]+ (Giuffrida et al. 2006), loss of toluene (M-92) (Britton et al. 2012) or a combination of toluene and water loss (M-110) are also encountered as in-source fragments in carotenoids analysis (Bijttebier et al. 2013). The high molecular weight of the three separated carotenoid fractions of EGY7 pigment extract indicated that they are esterified carotenoids. The molecular ion peak [MH+] of the first fraction (at 851) was similar to antheraxanthin monoester found in orange peels, it demonstrated a fragment at 531, equivalent to the loss of two water molecules and an alkyl C18 group plus an alkyl moiety. The additional loss of toluene resulted in a peak with a molecular weight of 438 (Murador et al. 2019). The second fraction represented astaxanthin monoester (M-C18:1) at molecular ion peak 861 similar to that produced by *Haematocccus pluvialis* (Todorović et al. 2021), while the third fraction was identified as zeaxanthin monoester (M-C9:1) at molecular ion peak 719 and a fragment at 657.4 corresponding to the molecular weight of zeaxanthin (Maoka 2023) (Fig. 5).

Generally, the effect of the imbalanced expression of proapoptotic and pro survival modulator of BCL-2 family proteins on tumor development and the resistance of malignant cells to anti-cancer agents is well studied. Thus, BCL-2 family members and their regulators are desirable targets for the development of anticancer agents. In this study, the efficacy of *Paracoccus* sp. EGY7 carotenoids against triple-negative cancer cell line (MDA-MB231) was investigated through estimating gene expression of *BAX* and *BCL-2* using qPCR. In addition, western blotting of the two proteins was carried out to assess protein expression. Our findings revealed that the extracted carotenoids significantly reduced cell viability and metastasis as verified by neutral red uptake and migration test. Additionally, they increased BAX expression, and effectively suppressed BCL-2 expression at both the mRNA and protein levels. These results are supported by other reports highlighting the superiority of xanthophylls as anti-cancer agents.

McCall et al. 2018 found that applying astaxanthin significantly reduced proliferation rates and inhibited the migration of breast cancer cells when compared to normal breast epithelial cells in the control group (McCall et al. 2018). Another study on colon cancer cell line (LS-180) showed that treatment with 100 and 150 µM concentrations of astaxanthin increased *BAX* gene expression compared to the control group, though this increase was not statistically significant (*P* > 0.05). However, *BCL-2* gene expression was significantly suppressed at these concentrations (*P* < 0.01) (Hormozi et al. 2019). Lutein was also shown to suppress antioxidant defense and cell survival markers, leading to apoptosis. This was evidenced by increased caspase-3 activity and decreased expression of BCL-2 and poly-ADP ribose polymerase. These findings underscore lutein’s role as a potent inhibitor of human breast cancer cell growth, facilitating cell death partly through the modulation of antioxidant defense response-related signaling markers (Kavalappa et al. 2021). Furthermore, additional research suggested that lutein could be a promising candidate for breast cancer chemoprevention, with HES1 potentially playing a crucial role in mediating lutein’s suppression of hypoxia-induced, ROS-driven breast cancer progression (Li et al. 2018). The substantial impact of pigment concentration on the expression of BAX and BCL-2 was investigated at two different concentrations 600 µg & 1200 µg. The analysis of BAX protein expression indicated significant increases with higher treatment concentration. Meanwhile, BCL-2 protein expression shows a consistently decreased with increasing treatment concentrations, highlighting pronounced reductions in the 1200 µg and 600 µg groups. These results indicate that higher concentrations of the pigment may promote apoptosis by shifting the balance towards pro-apoptotic BAX over anti-apoptotic BCL-2. This shift could lead to increased cell death (Qian et al. 2022), potentially offering a therapeutic avenue for targeting cancer cell survival mechanisms.

In our study the gene expression analysis revealed a decrease in *BCL-2* gene expression and an increase in *BAX* gene expression. These findings underscore the substantial impact of pigment concentrations, particularly at 1200 µg and 600 µg, on the *BCL-2/BAX* ratio gene expression in MDA-MB-231 cells.

Several other mechanisms are displayed by carotenoids against different cancer cel lines. For instance, lutein extracted from different vegetables, demonstrated G0/G1 phase arrest in lung cancer and increased survival in mouse models (Zhang et al. 2023). Astaxanthin reduces proliferation in leukemia, breast, and colon cancers by downregulating key proteins like ERK and p27 (Kim et al. 2016; Liu et al. 2016). Additionally, fucoxanthin exhibits broad-spectrum anticancer activity by suppressing cyclin and CDK expression in gastric and bladder cancers and modulating JAK/STAT pathways in dose dependent manner (50–75 µM) (Wang et al. 2014; Yu et al. 2011). These mechanisms underscore the diverse roles of carotenoids in targeting cancer cell proliferation and their therapeutic potential (Baeza-Morales et al. 2024).

Generally, Molecular docking tools are used for forecasting how small ligand molecules will interact with the proper binding sites of the desired protein. Molecular docking analysis is commonly applied to estimate the binding affinity of different anticancer agents with antiapoptotic protein BCL-2 (Poustforoosh et al. 2022). An in-silico molecular docking was conducted by Suganya and Anuradha, to evaluate the interaction of astaxanthin and sorafenib, a positive control drug with growth factor receptors such as VEGFR2 and EGFR, as well as apoptotic proteins like BCL-2, Caspase 3, and Caspase 9 to elucidate their anticancer mechanisms in hepatocellular carcinoma (HCC) cell line. In the analysis of the binding interactions with the BCL-2 protein, the ΔG for astaxanthin and sorafenib revealed notable differences. Astaxanthin demonstrated a ΔG of -4.75 kcal/mol, indicating a moderate binding affinity with BCL-2. In contrast, sorafenib exhibited a stronger binding affinity with a ΔG of -5.53 kcal/mol (Suganya and Anuradha 2019).

On the other hand, our molecular docking analysis revealed that zeaxanthin exhibits a higher affinity and lower ΔG compared to obatoclax, the reference drug for BCL-2. The interaction between Bcl-2 and zeaxanthin indicated a stronger and more stable binding with ΔG of -9.773241. In contrast, obatoclax showed a moderately strong but less favorable interaction with BCL-2 with ΔG of -7.419345. These results indicate that zeaxanthin exhibits greater affinity and a lower ΔG compared to astaxanthin, sorafenib, and obatoclax when interacting with BCL-2. This suggests that zeaxanthin holds greater potential as an anticancer agent targeting this protein.

This study employs a comprehensive methodology, utilizing qPCR, Western Blotting, and Molecular Docking to analyze the effects of *Paracoccus* sp. EGY7 carotenoids on the triple-negative breast cancer (TNBC) cell line MDA-MB-231. This multi-faceted approach ensures a thorough examination of gene and protein expression as well as molecular interactions. The novelty of exploring *Paracoccus* sp. EGY7 carotenoids on TNBC, an aggressive cancer subtype with limited treatment options, adds valuable knowledge to the field. However, the study’s in vitro nature may not fully translate to clinical settings, and focusing on a single cell line limits its scope. Challenges in isolating specific carotenoids could impact reproducibility. The technical variability in qPCR and Western Blotting may introduce inconsistencies. The complexity of cancer cell signaling complicates identifying direct effects, and the absence of in vivo data limits the assessment of therapeutic potential in a physiological context.

Future research should include in vivo studies to validate effects in animal models and expand to multiple TNBC cell lines for greater generalizability. Standardizing carotenoid isolation and characterization will improve reproducibility. Investigating synergistic effects with existing therapies and detailed signaling pathways will enhance understanding. Finally, initiating clinical trials will be crucial for translating these findings into potential treatments for TNBC patients.

In conclusion, the carotenoid pigment from *Paracoccus* sp. EGY7 exhibits significant anti-cancer properties, underscoring its potential as a therapeutic agent against breast cancer. This potential is evidenced by its ability to modulate the expression of the pro-apoptotic gene *BAX* and the anti-apoptotic gene *BCL-2*, thereby inducing apoptosis in cancer cells. Furthermore, the carotenoid pigment effectively inhibits the migration of breast cancer cells, a critical factor in preventing metastasis and disease progression.

**Table 1:** Analysis of the HPLC chromatogram of the extracted carotenoids showing retention time (TR) and maximum absorption wavelength (λmax) of the five separated fractions. LC/ESI-MS/MS Mass spectra of antheraxanthin monoester, astaxanthin monoester, zeaxanthin monoester.

Peak number	T_*R*_^a^ (min)	λ max (NM)	[M + H]^+^ (m/z)	Carotenoid Identificaton	Formula	Compound (%)
1	5	475	851	Antheraxanthin monoester	M-C18:0	11.7
2	5.8	ND	ND	-	-	4.7
3	9.7	470	861.6	Astaxanthin monoester	M-C18:1	23
4	16.9	ND	ND	-	-	12.2
5	26	458	719.77	Zeaxanthin monoester	M-C9:1	48.3

ND: Not Detected.

**Table 2:** Typical ions detected in positive ion mode (ESI- MS) and products ions in ESI MS/MS of Paracoccus sp. EGY7 carotenoid components.

Carotenoid	[M + H]^+^ (m/z)	Fragment ions (m/z)	References
Antheraxanthin-C18:0	851	531.3 [M + H–C18:0–18 − 18]^**+.**^ 438 [M–C18:0–92– 18 − 18]^**+.**^	42
Astaxanthin-C18:1	861.6	719 [M + H – 106–18 − 18]^**+.**^	43
Zeaxanthin-C9:1	719.77	567 [M – C9:1]^**+.**^	44

## Electronic supplementary material

Below is the link to the electronic supplementary material.

Supplementary Material 1

## Data Availability

All data generated or analysed during this study are included in this published article.
