# Supplementary material for: Impact of Paracoccus sp. EGY7 carotenoids on triple-negative breast cancer cells: invitro study
Source: AMB Express. 2025 Feb 7;15:21. doi: 10.1186/s13568-025-01825-5 (PMC11805741; doi:10.1186/s13568-025-01825-5)
Supplement: Supplementary file 1 — Supplementary Material 1 [file 13568_2025_1825_MOESM1_ESM.docx]

**Section 1: Isolation and purification of pigment producing bacteria**

Bacterial isolation was carried out from different soil samples. The collected samples were first activated on PY medium for 18 h. The activated cultures were serially diluted using 0.9% saline, spread on PA plates, then incubated at 30 ^o^C. The colored colonies were picked up, purified, and stored at -20 ^o^C in 20% glycerol.

The selected isolates were examined morphologically and microscopically, and were further subjected to molecular identification using the 16S *rRNA* approach according to the previously reported method (Eden et al., 1991). Genomic DNA was extracted according to the manufacturer’s instructions using the DNA miniprep kit (D3024, ZYMO).

The polymerase chain reaction technique (PCR) was used to amplify the whole length of the 16S *rRNA* gene using the universal primers F27 and R1492. The amplified PCR product was purified using the PCR Clean Up Kit SV-Wizard (ZYMO Co., USA), and its sequence was determined through the automated fluorescent DNA sequencer. The obtained nucleotide sequence was processed using BioEdit software, the curated sequence was then analyzed using the BLAST n tool provided by NCBI. The phylogenetic tree was created using MEGA X software to estimate the taxonomic relationship of the isolate with closely related hits.

**Section 2: Reverse transcription quantitative polymerase chain reaction (RT qPCR)**

RT qRCR was used for the quantitative determination of the relative expression of *BCL-2* and *BAX* genes. MDA-MB-231 cells were cultured in 6-well plates then treated with either 0, 600 or 1200 µg of pigment and DMSO for 48 hrs before they were trypsinized, washed by PBS, centrifuged and the supernatant was decanted. Total RNA was isolated using Fast HQ Extraction kit (Intron Inc., cat#17213) according to manufacturer’s instructions. The concentration and purity of isolated was determined by measuring absorbance ratio at 260/280 nm using a Thermo Scientific ND2000 Nanodrop Spectrophotometer. Freshly isolated total RNA to complementary DNA (cDNA) using SensiFAST^TM^ cDNA synthesis Kit (Meridian Life Science Inc, USA, cat #BIO-65053) on an Applied Biosystems GeneAmp PCR System 9700 N8050200 thermal cycler with the following settings: 25 °C for 10 min, 42 °C for 15 min, 85 °C for 5 min, and 4 °C on hold. cDNA was stored at −20 until qPCR experiments. The RT qPCR reaction was performed on CFX Connect Real-Time PCR System (BioRad Inc., USA) using SensiFAST SYBR Mix (2X) (Meridian Life Science Inc, USA, cat #BIO-98005). The primer sequences were as follows: BCL2 forward, 5' ATCGCCCTGTGGATGACTGAGT 3' and reverse, 5’ GCCAGGAGAAATCAAACAGAGGC 3', BAX forward, 5' TCAGGATGCGTCCACCAAGAAG 3' and reverse, 5' TGTGTCCACGGCGGCAATCATC 3' and GAPDH forward, 5’ GTCTCCTCTGACTTCAACAGCG 3’ and reverse, 5’ ACCACCCTGTTGCTGTAGCCAA 3’. The cycling conditions were 10 min at 95˚C for initial activation followed by 40 cycles of 5 sec at 95˚C, 10 sec at 58˚C and 20 sec at 72˚C. The obtained results were presented as the average fold change of target gene in test to control group using the 2^-ΔΔCt^ formula.

**Section 3: Western Blotting**

BCl-2 and BAX proteins were quantified using western blotting technique. Total protein extraction was done using ReadyPrepTM protein extraction kit (Bio-Rad Inc., cat#163-2086). The cells were harvested and 50 µl of cell pellet was suspended in 1 ml of freshly prepared tributylphosphine (TBP). The cell suspension was then sonicated for a total of 120 sec then centrifuged at 16,000 xg for 20 min at 4°C. The pellet was discarded and the supernatant containing the soluble protein was collected and kept on the ice for further analysis. Total protein was quantified using Bradford assay kit (Bio basic Inc., Canada) according to manufacturer’s instructions. A sample volume equivalent to 20 μg of protein was loaded with an equal volume of 2x Laemmli sample buffer containing 4% SDS, 10% 2-mercaptoehtanol, 20% glycerol, 0.004% bromophenol blue and 0.125 M Tris HCl (pH 6.8), boiled at 95°C for 5 min then loaded on 15% SDS-PAGE bis-tris gels (Bio-Rad inc., cat #161-0181). Separated proteins were transferred to nitrocellulose membranes using 25 mM Tris and 190 mM glycine and 20% methanol transfer buffer in wet transfer system. Following the band transfer, the membrane was blocked in 25 ml of TBST buffer (20 mM Tris pH 7.5, 150 mM NaCl, 0.1% Tween 20) with 3% bovine serum albumin for 1 hr at room temperature. The membrane and primary antibody (Novus Biologicals Inc., USA) were incubated in 10 ml primary antibody dilution buffer with gentle agitation overnight at 4°C and washed with TBST 3-5 times for 5 min each. After incubation with secondary horseradish peroxidase-conjugated antibody (at 1:2000) (Novus Biologicals Inc., USA) for 1 hr at room temp, the membrane was rinsed 3–5 times for 5 min with TBST, proteins were detected by incubating the membrane in 10 ml Clarity^TM^ Western ECL substrate (Bio-Rad Inc., cat#170-5060), 5 ml Reagent A, 5 ml Reagent B with gentle agitation for 1 min at room temperature. The chemiluminescent signals were captured using a CCD camera-based imager. Image analysis software was used to read the band intensity of the target proteins against control sample beta actin (housekeeping protein) by protein normalization on the ChemiDoc MP imager.


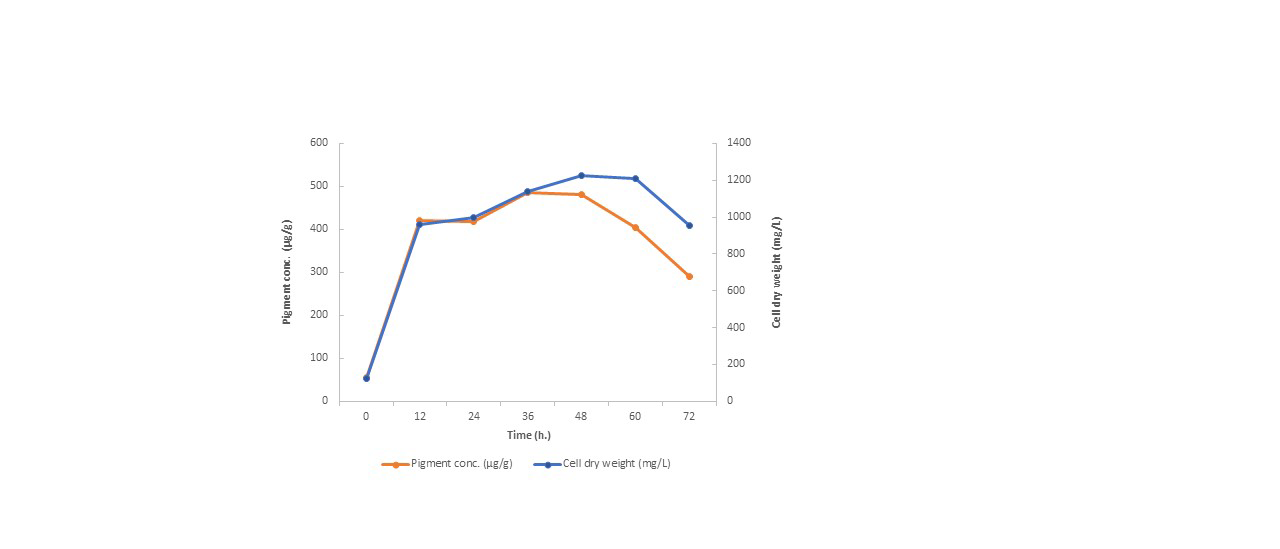


**Figure 1S:** Biomass and pigment accumulation pattern of the *Paracoccus* sp. EGY7 strain cultivated on PYT medium during 3 days of incubation.


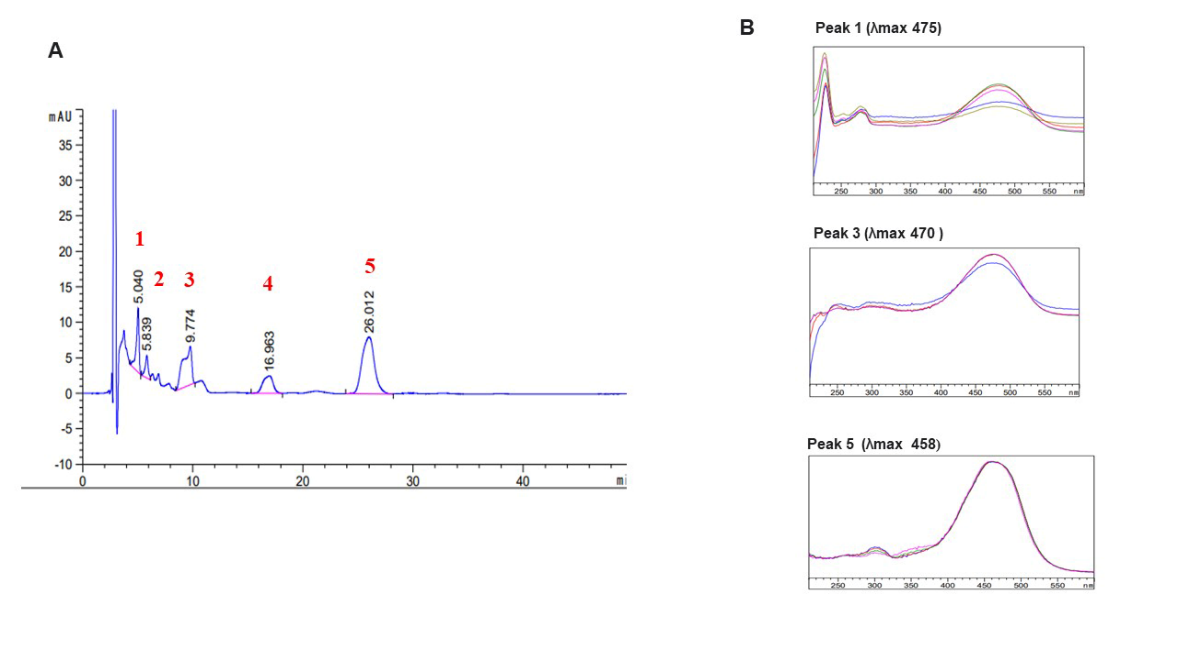


**Figure 2S:** A) chromatogram of the extracted carotenoids shows five eluted peaks. B) UV/Vis spectrum characteristics of peaks 1, 3, and 5 of *Paracoccus* sp. EGY7 carotenoids.


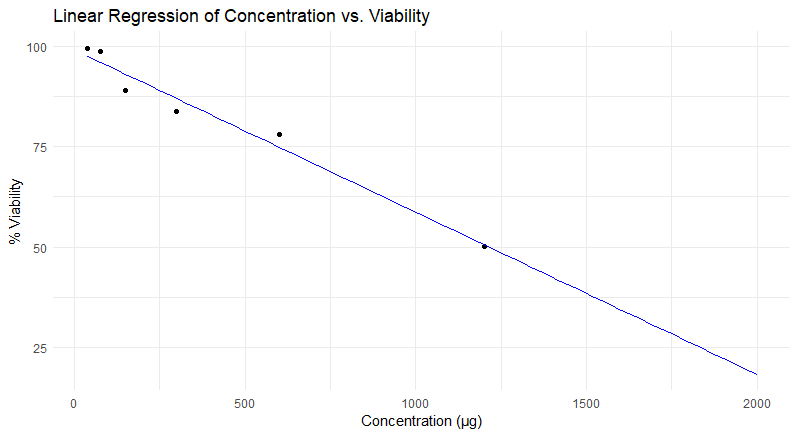


**Figure 3S:** Linear regression of pigment concentration vs. viability of tumor cells


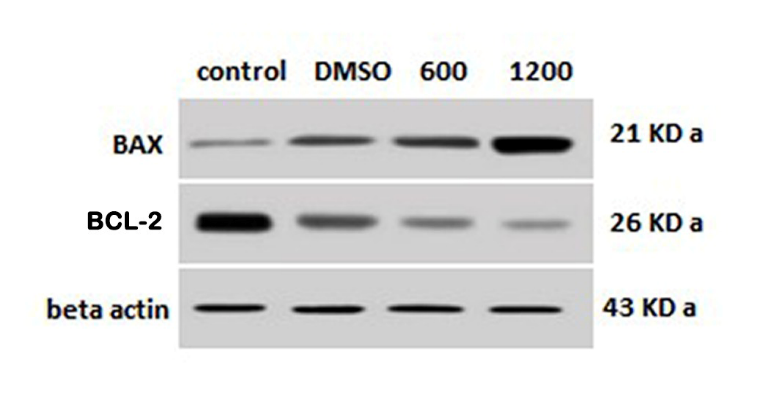


**Figure 4S:** BAX and BCL-2 protein expression bands

**Table 1S:** Tukey Post Hoc Analysis of BCL-2/BAX Ratio Gene Expression in MDA-MB-231 Cells Treated with *Paracoccus* sp. EGY7 Carotenoid Pigment

| **Comparisons** | **p-value** | **Significant Level** |
| --- | --- | --- |
| **1200 µg vs. Control** | 0.0019536 | Significant |
| **1200 µg vs. DMSO** | 0.0015685 | Significant |
| **600 µg vs. Control** | 0.0194848 | Significant |
| **600 µg vs. DMSO** | 0.0149616 | Significant |
| **DMSO vs. Control** | 0.9970772 | Not Significant |
| **600 µg vs. 1200 µg** | 0.3189472 | Not Significant |

**Table 2S:** Tukey Post Hoc Analysis of BAX and BCL-2 Protein Expression Levels in MDA-MB-231 Cells Treated with *Paracoccus* sp. EGY7 Carotenoid Pigment

| **Comparisons** | **p-value** | **Significant Level** |
| --- | --- | --- |
| **BAX Protein Expression** | | |
| **1200 µg vs. 600 µg** | 0.0006 | Significant |
| **1200 µg vs. Control** | 0.0001 | Significant |
| **1200 µg vs. DMSO** | 0.0001 | Significant |
| **600 µg vs. Control** | 0.0008 | Significant |
| **600 µg vs. DMSO** | 0.0012 | Significant |
| **DMSO vs. Control** | 0.6405 | Not Significant |
| **BCL-2 Protein Expression** | | |
| **1200 µg vs. 600 µg** | 0.0157 | Significant |
| **1200 µg vs. Control** | 0.00005 | Significant |
| **1200 µg vs. DMSO** | 0.00008 | Significant |
| **600 µg vs. Control** | 0.0001 | Significant |
| **600 µg vs. DMSO** | 0.0002 | Significant |
| **DMSO vs. Control** | 0.0508 | Not Significant |

**Table 3S:** Scratch reduction rate

| **Treatment** | **Reduction rate 0 – 24 hr (%)** | **Reduction rate 0 – 48 hr (%)** |
| --- | --- | --- |
| **Control** | 71.67 | 95.67 |
| **DMSO** | 44.98 | 86.61 |
| **600 µg** | 37.50 | 79.17 |
| **1200 µg** | 12.50 | 53.50 |

**Table 4S:** Molecular docking analysis results

| **Docking** | **ΔG (kcal/mol)** | **FullFitness value (kcal/mol)** |
| --- | --- | --- |
| **BCL2-ZXT** | -9.773241 | -2218.46 |
| **BCL2- obatoclax** | -7.419345 | -2203.04 |

References:

Eden, P. A., Schmidt, T. M., Blakemore, R. P., & Pace, N. R. (1991). Phylogenetic analysis of Aquaspirillum magnetotacticum using polymerase chain reaction-amplified 16S rRNA-specific DNA. *International journal of systematic and Evolutionary microbiology*, *41*(2), 324-325.
